# Blackberry Leaves as New Functional Food? Screening Antioxidant, Anti-Inflammatory and Microbiological Activities in Correlation with Phytochemical Analysis

**DOI:** 10.3390/antiox10121945

**Published:** 2021-12-04

**Authors:** Magdalena Paczkowska-Walendowska, Anna Gościniak, Daria Szymanowska, Dominik Szwajgier, Ewa Baranowska-Wójcik, Piotr Szulc, Dagna Dreczka, Marek Simon, Judyta Cielecka-Piontek

**Affiliations:** 1 Department of Pharmacognosy, Poznan University of Medical Sciences, Rokietnicka 3, 60-806 Poznan, Poland; annagos97@gmail.com (A.G.); jpiontek@ump.edu.pl (J.C.-P.); 2 Department of Biotechnology and Food Microbiology, Poznan University of Life Sciences, Wojska Polskiego 48, 60-627 Poznan, Poland; darszy@up.poznan.pl; 3 Department of Biotechnology, Microbiology and Human Nutrition, University of Life Sciences in Lublin, Skromna 8, 20-704 Lublin, Poland; dominik.szwajgier@up.lublin.pl (D.S.); ewa.baranowska@up.lublin.pl (E.B.-W.); 4 Department of Agronomy, Poznan University of Life Sciences, Dojazd 11, 60-632 Poznan, Poland; piotr.szulc@up.poznan.pl; 5 Department of Rehabilitation and Physiotherapy, Poznan University of Medical Sciences, 28 Czerwca 1956r. 135/147, 61-545 Poznan, Poland; dagna.dreczka@ump.edu.pl; 6 Department of Pathophysiology, Poznan University of Medical Sciences, Rokietnicka 8, 60-806 Poznan, Poland; msimon@ump.edu.pl

**Keywords:** blackberry, leaves, Loch Tay variety, agri-food waste, antioxidant activity, anti-inflammatory activity, microbiological activity

## Abstract

Blackberry fruits are recognized as functional foods while blackberry leaves are outside this classification and they also contain active compounds with health-promoting potential. Therefore, the aim of this study was the phytochemical analysis of blackberry leaves of varieties (Chester, Loch Ness, Loch Tay and Ruczaj) and screening of their biological activity (antioxidant potential, possibility of inhibition of enzymes, anti-inflammatory and microbial activity). The following compounds from selected groups: phenolic acids (caffeic acid, ellagic acid, gallic acid, syringic acid), flavonols (quercetin, kaempferol) and their glycosides (rutin, isoquercetin, hyperoside) and flavon-3-ols (catechin, epicatechin) were chromatographically determined in the aqueous and hydroalcoholic leaves extracts. All tested blackberry leaves extracts showed antioxidant effects, but the highest compounds content (TPC = 101.31 mg GAE/g) and antioxidant activity (e.g., DPPH IC_50_ = 57.37 μg/mL; ABTS IC_50_ = 24.83 μg/mL; CUPRAC IC_50_ = 62.73 μg/mL; FRAP IC_50_ = 39.99 μg/mL for hydroalcoholic extracts) was indicated for the Loch Tay variety. Blackberry leaf extracts’ anti-inflammatory effect was also exceptionally high for the Loch Tay variety (IC_50_ = 129.30 μg/mL), while leaves extracts of the Loch Ness variety showed a significant potential for microbial activity against *Lactobacillus* spp. and *Candida* spp. Summarizing, the best multidirectional pro-health effect was noted for leaves extracts of Loch Tay variety.

## 1. Introduction

At present, foods are intensively tested for additional physiological benefits that can reduce the risk of chronic disease or otherwise optimize health. With documented health benefits, such foods can be referred to as functional foods [1]. One such example could be blackberries, which are recognized worldwide as a functional food and their high interest is connected with the high content of polyphenols compounds, which greatly contribute to their organoleptic properties and health benefits [2].

In recent years, Europe has become one of the world leaders in the production of blackberries (*Rubus fruticosus*). The crops development is promoted by favorable climatic and soil conditions as well as the cultivators experience [3]. Farmers have generated a number of cultivars of *R. fruticosus* using traditional breeding procedures, which differ in fruit firmness, shape, size, flavor, color, weight, yield, ripening season, nutritional contents, and resistance to pests. The most well-known cultivars include Jumbo, Chester, Bartin, Ness, Bursa 1, Bursa 2, Bursa 3, Arapaho, Navaho, Thornfree, Chester Thornless, Dirksen Thornless, Cacanska Bestrna, Loch Ness, Cherokee, and Black Satin [4]. Notably, despite the great interest in blackberry fruits, also their leaves have been used in traditional remedies. Leaves are byproducts of berry cultivation and are often treated as waste when growing fruit. The use of agri-food waste products, such as leaves, seeds, bark and branches, is not a new approach, but becoming an increasingly important alternative to obtaining raw materials with significant economic potential [5,6,7,8,9,10]. Therefore, it is also worth looking at the content of bioactive compounds in the leaves and their application.

Phenolic acids (hydroxybenzoic and hydroxycinnamic acids and their derivatives), flavonoids, such as flavonols, flavanols, and anthocyanins, and tannins are all examples of polyphenols found in blackberries [2]. The compositions of the blackberry leaves are listed in Table 1.

Anti-microbial, antioxidant, anti-inflammatory, and anti-cancer activities are just some of the multiple biological effects attributed to flavonoids and other phenolic compounds. The majority of pharmacological effects may be traced back to phenolic compounds, which aid in the scavenging of free radicals that cause a variety of clinical and metabolic diseases [4]. Blackberry leaves provide extracts used in traditional medicine as a mouthwash against thrush, gum inflammations, mouth ulcers, and sore throat [11]. Finally, they have long been utilized to treat a variety of respiratory issues [11]. The tannins in blackberry leaves are responsible for some beneficial effects for diarrhea treatment and similar enteric disorders and as an astringent. However, tannins can cause liver damage if ingested in large amounts over long periods [12]. Blackberry leaves are known to eliminate free radicals that damage cells, and as antioxidants, they can help prevent lifestyle diseases. They also aid in the protection and strengthening of immunity, as well as lowering the risk of cancer [13]. 

Blackberry is one of the highest sources of anthocyanins, flavonol glycosides, and other phenols among common fruits and vegetables, contributing to its strong antioxidant potential. However, there is a scarcity of information on the chemical makeup of *Rubus* leaves. Despite the fact that the phenolic content of the leaves is affected by environmental factors and harvest maturity, it is critical to understand the chemical composition and anti-oxidant properties of the various *Rubus* species in order to use them selectively in the pharmaceutical and food industries [14].

To the best of our knowledge, no comparative studies have been carried out on the chemical composition of leaves of the new *Rubus* varieties. Consequently, the aim of the research was to identify polyphenolic compounds and their content, as well as their biological properties, including antioxidant, anticholinesterase, anti-inflammatory, and microbiological activity, which can be extremely valuable both in human health but also as an economic use of agri-food waste.

## 2. Materials and Methods

### 2.1. Chemicals and Reagents

Standards of phenolic acids: caffeic acid, ellagic acid, gallic acid, syringic acid; flavonols: quercetin, kaempferol, and their glycosides: rutin, isoquercetin, hyperoside; flavon-3-ols: catechin, epicatechin were obtained from Sigma-Aldrich (Poznan, Poland), as well as all reagents for antioxidant and anti-hyaluronidase activity assays: Folin-Ciocalteu’s phenol reagent, sodium carbonate, 2,2-Diphenyl-1-picrylhydrazyl (DPPH), potassium persulfate (K_2_S_2_O_8_), 2,2′-Azino-bis(3-ethylbenzothiazoline-6-sulfonic acid) diammonium salt (ABTS, C1_8_H_24_N_6_O_6_S_4_), neocuproine, ammonium acetate, copper(II) chloride (CuCl_2_·H_2_O), sodium acetate trihydrate (CH_3_COONa·3H_2_O), 2,4,6-tris(2-pyridyl)-1,3,5-triazine (TPTZ, C18H12N6), iron(III) chloride hexahydrate (FeCl_3_·6H_2_O), sodium chloride, bovine serum, hexadecyltrimethylammonium bromide (CTAB), and hyaluronic acid (HA). The following chemicals from Sigma-Aldrich (Poznan, Poland) were used: DMSO D4540, AChE C3389, BChE C7512, ATChI A5751, BTCh B3128, DTNB D8130, donepezil D6821, neostigmine N2001, magniflorine SMB00377, rivastigmine SML0881, eserine E8375, neocuproin N1501, CuCl2 307483, TPTZ 93285, Trolox 238813, fluorescein 46955, CoF_2_ 236128, linoleic acid L1376, Tween20 P1379, β-carotene C9750, Tween 80 P1754, ascorbic acid A92902, glutathione reductase G3664, glutathione (GSH) G4251, glutathione peroxidase G6137, nicotinamide adenine dinucleotide phosphate (NADPH) N5130, ethylenediaminetetraacetic acid (EDTA) E9884, glutathione disulfide (GSSG) G4626, superoxide dismutase (SOD) S5395, nitrobluetrazolium N6639, xanthine X0626, and xanthine oxidase X4875. 2,2’-Azobis(2-amidinopropane) dihydrochloride (AAPH) was from Acros Organics (Poznan, Poland) (401560250), cyclooxygenase-2 (COX-2, human recombinant, 60122) and COX-2 activity assay kit (760151) were from Cayman Chemicals (Ann Arbor, Michigan, USA). Buffer salts, solvents and other reagents were from Sigma Aldrich and were at least of analytical grade. HPLC grade acetonitrile and methanol were obtained from Merck (Warsaw, Poland). High-quality pure water and ultra-high-quality pure water were prepared by using a Direct-Q 3 UV Merck Millipore purification system (Merck, Darmstadt, Germany).

### 2.2. Preparation and Analysis of Rubi Folium Extract

#### 2.2.1. Plan Material

Blackberry leaves for the study were derived from the “Szynsad” Orchard Farm in Dąbrówka Nowa near Grójec Mazowieckie, Poland (51°47′01″ N 20°43′04″ E) in 2020. It was a plantation in the third year of cultivation. The basis of fertilization was chemical soil analysis for the assessment of basic nutrient contents. There was an abundance of soil phosphorus, potassium and magnesium in the spring (start of vegetation). At that time they were at the following levels: 69.0 mg P·kg^−1^ soil; 105.0 mg K·kg^−1^ soil; and 39.0 mg Mg·kg^−1^ soil, respectively. The mineral fertilization program was as follows: start of vegetation NPK 12-11-18+Mg, the start of flowering NK 14-24+Ca, the development of green parts of the plant NPK 14-1.5-7+Mg+Fe, and flowering and fruit development Ca + K. Disease control on the blackberry plantation was carried out in accordance with the Plant Protection Program.

Leaf-blades of the following varieties were collected for the study:

− *Rubus* Chester—thornless variety, the mature shrub grows up to a height of 1.5 m high; the variety is susceptible to anthracnose, gray mold and verticillium wilt.
− *Rubus* Loch Ness—thornless variety, the mature shrub grows up to a height of 5 m, thermal and soil requirements of this variety are low.
− *Rubus* Loch Tay—thornless variety, the mature shrub grows up to a height of 1.5 m, fruits on two-year-old shoots, average thermal and soil requirements.
− *Rubus* Ruczaj—thornless variety, the mature shrub grows to a height of approximately 2 m, medium thermal and soil requirements, susceptible to gray mold, medium thermal and soil conditions.

#### 2.2.2. Extract Preparation

100.0 mg of plant raw material was extracted with 5.0 mL of water or methanol and distilled water (7:3 *v*/*v*) mixture at the temperature of 50 °C in an ultrasonic bath: three times for 30 min. Extracts were concentrated to 10.0 mL to yield a stock solution (10.0 mg dry leaf/mL).

#### 2.2.3. Determination of Active Compounds Content in Extracts

The concentrations of active substances were measured by using the HPLC-DAD (Dionex Thermoline Fisher Scientific, Waltham, MA, USA) with Chromeleon software version 7.0. Separations were performed on a LiChrospher RP-18 column, 5 μm particle size, 250 mm × 4 mm (Merck, Darmstadt, Germany). The detection was done with a diode array detector at a wavelength maxima (*λ*_max_) of 270 and 360 nm, depending on active compounds. The mobile phase was composed of formic acid 0.1% in water (A) and acetonitrile (B) with a gradient elution: 0–35 min, 2–20% B; 35–55 min, 20–70% B; 55 min, 2% B; 55–60 min, 2% B, with mobile phase flow set at 1.0 mL/min. The column temperature was kept at 30 °C.

The presence of 11 active compounds (phenolic acids: caffeic acid, ellagic acid, gallic acid, syringic acid; flavonols: quercetin, kaempferol, and their glycosides: rutin, isoquercetin, hyperoside; flavon-3-ols: catechin, epicatechin) in the extracts was confirmed by comparison of retention time and UV spectra of particular substances; whereas, the quantitative assessment of the content included 7 actives (caffeic acid, ellagic acid, quercetin, kaempferol, rutin, hyperoside, epicatechin). 

In terms of selectivity, linearity, intra- and inter-day accuracy, limits of detection (LOD), and quantitation, the HPLC-DAD method was validated according to the International Conference on Harmonization Guideline Q2 (LOQ) [15].

#### 2.2.4. Total Phenolic Content (TPC)

Kikowska et al. [16] described the Folin-Ciocalteu method for determining TPC. To 25 µL of the extracts or gallic acid solution (in concentration range 6.25–100 μg/mL), 200 µL of distilled water, 15 µL of Folin-Ciocalteu reagent and 60 µL of 20% sodium carbonate solution were added. In dark conditions, the plate was shaken for 5 min at room temperature at 600 rpm, then incubated for another 25 min at room temperature. Six replicates were used in the analysis. The total gallic acid content in the produced extracts was estimated using the standard substance’s calibration curve and represented as milligrams of gallic acid equivalents (GAE) per 1 g of plant material.

#### 2.2.5. Antioxidant Activity

##### Assay with 2,2-Diphenyl-1-picrylhydrazyl (DPPH)

A total of 25 μL of extracts were combined with 175 μL of DPPH solution (3.9 mg/50 mL methanol). The reaction mixture was shaken and incubated at room temperature for 30 min in the dark. The absorbance of 25 μL of water or a 3:7 *v*/*v* water-methanol mixture and 175 μL of methanol was measured at 517 nm against a blank (25 μL of water or a 3:7 *v*/*v* water-methanol mixture and 175 μL of methanol) [10]. Six replicates were used in the analysis. The percentage of DPPH scavenging activity was estimated using the following formula:

$$DPPH_{\mathrm{scavenging}\;\mathrm{activity}\;}\left(\%\right)=\frac{A_{0}-A_{1}}{A_{0}}\times 100\%$$

where *A*_0_ is the absorbance of the control, and *A*_1_ is the absorbance of the sample. 

The IC_50_ value, which corresponds to the concentration of the extract required to block radical production by 50%, was calculated from the results.

##### 2,2-Azino-bis(3-ethylbenzothiazoline-6-sulfonic Acid) (ABTS) Radical Cation-Based Assays 

A volume of 200 µL of the ABTS solution (0.0384 g ABTS dissolved in 10 mL of aqueous 2.45 mM potassium persulfate solution left for 24 h) was added to 50 µL of the extract (in concentration range 0.08–2.50 mg/mL) and then incubated in the dark condition for 10 min at room temperature. The absorbance was then measured at λ = 734 nm against a blank (50 µL of water or a 3:7 *v*/*v* water-methanol mixture and 200 µL of water). Six replicates were used in the analysis. The equation was used to calculate the ability to remove free radicals (%):

$$ABTS_{\mathrm{scavenging}\;\mathrm{activity}\;}\left(\%\right)=\frac{A_{ABTS}-A_{s}}{A_{ABTS}}\times 100\%$$

where *A_ABTS_* is the absorbance of ABTS cation radical solution, and *A_S_* is the sample absorbance.

The obtained IC_50_ values correspond to represent the quantity of antioxidant required to inhibit 50% of the radical.

##### Oxygen Radical Absorbance Capacity (ORAC) Assay 

A sample of 10 μL was tested by combining with 170 μL of fluorescein (0.00020941 mg fluorescein/10 mL, 75 mM phosphate buffer, pH 7.4) and incubated 20 min at 37 °C. Then, 20 μL AAPH (0.14248 mg AAPH/mL buffer) was added and the fluorescence was read (excitation at 485 nm and emission at 520 nm) at the start and after 1 min, with continual shaking during the whole reaction, until stability. The blank sample contained phosphate buffer, instead of the sample. In addition, the background from the samples was measured (a mixture containing the studied sample and DDI water only). The activity was determined using a 50 μM stock solution and 12 dilutions to obtain the Trolox equivalents [17].

##### Effect on Superoxide Dismutase (SOD) Activity

A sample of 0.05 mL was combined with 10 μL SOD (0.24 U), 160 μL nitrobluetatrazolium solution (0.0025 M), 205 μL phosphate buffer (0.2 M, pH 7.5), 30 μL xanthine (150 mM in 1 M NaOH) and 0.01 mL xanthine oxidase (0.065 U). The difference in absorbance at 550 nm in tested samples vs. controls without studied samples was obtained after 20 min of incubation, and the effect on the enzyme was calculated using the following equation [18]:

$$Inhibition\left(\%\right)=100-100\times \frac{A_{30min}-A_{0min}}{A_{control\;30min}-A_{control\;0min}}$$

##### Cupric Ion Reducing Antioxidant Capacity (CUPRAC) Assay 

A volume of 150 µL CUPRAC reagent (equivalent volumes of 7.5 mM neocuproine solution in 96% ethanol, acetate buffer (pH = 7.0), and 10 mM CuCl_2_·H_2_O solution) was added to 50 µL extracts in a well of a 96-well plate for the CUPRAC assay [10]. The plate underwent shaking for 5 min before being incubated at room temperature for 25 min in the dark. The absorbance was then measured at 450 nm. Six replicates of the analysis were carried out. The results were expressed as the IC_0.5_, which is the concentration of extract necessary to achieve an absorbance value of 0.5.

##### Ferric Ion Reducing Antioxidant Parameter (FRAP) Assay 

The FRAP assay was carried out according to Kikowska et al. procedure’s [16]. A volume of 25 µL of the extract (in the concentration range of 0.08–2.50 mg/mL) was added to 175 µL of the freshly made FRAP mixture (25 mL acetate buffer, 2.5 mL TPTZ solution, and 2.5 mL FeCl_3_·6H_2_O solution) and incubated in the dark for 30 min at 37 °C. The absorbance was then measured at 593 nm. Six replicates were used in the analysis. The IC_0.5_ value, which corresponded to the concentration of extract necessary to achieve an absorbance value of 0.5, was calculated.

##### Hydroxyl Radical Averting Capacity (HORAC) Assay 

Fluorescein solution (170 μL, 60 nM) was combined with the sample (0.01 mL) and incubated at 37 °C for 10 min. Then, to the tested sample, 10 μL of 27.5 mM H_2_O_2_ solution and 10 μL of CoF_2_·4H_2_O solution (230 μM, containing 1 mg of picolinic acid/mL) were added. The fluorescence was measured at the beginning and every 1 min after that until the process stabilized (excitation at 485 nm and emission at 520 nm) (typically 5–10 min). Instead of the sample, the blank sample contained phosphate buffer. In addition, the background from the samples was measured (a mixture containing the studied sample and DDI water only). The activity was measured in gallic acid equivalents (GAE), which were calculated using 15 gallic acid solutions (corresponding to 9.6–480.0 g of gallic acid/mL) as described previously [17].

##### Effect on Glutathione Reductase (GR) and Glutathione Peroxidase (GPx) Activity 

The effect on glutathione reductase (GR) was performed as follows. The sample (0.02 mL) was mixed with 10 µL EDTA solution, 12 µL GSSG solution, and incubated for 5 min at 25 °C before adding 4 µL NADPH solution (all reagents were diluted in 0.1 mM sodium phosphate buffer, pH 7.6) and recorded the first absorbance (340 nm). The reaction was then begun by adding 2 U glutathione reductase (2 µL/L), 177 µL/L of 0.1 mM sodium phosphate buffer, and measuring the absorbance after 5 min at 25 °C. The following were the reagent concentrations in the final mixture (805 µL/L): 10 mM GSSG, 0.5 mM EDTA, and 10 mM NADPH. Instead of the sample, a blank sample was created using the buffer, and the background was assessed (mixture containing studied sample and buffer only). In comparison to nmol of NADPH consumed/min in the blank (reagent) sample, one unit of enzyme activity was defined as nmol of NADPH consumed/min·mL sample. [19].

The effect on glutathione peroxidase (GPx) was performed as follows. A volume of 8 µL EDTA solution, 10 µL glutathione reductase (0.2 U), 4 µL GSH solution, 10 µL glutathione peroxidase (0.04 U), 22 µL H_2_O_2_, and 332 µL 50 mM sodium phosphate, pH 7.0) were mixed with the sample (0.020 mL). A volume of 4 µL of NADPH solution (N5130) was added to begin the reaction, and after 10 min of incubation at 25 °C, the decrease in absorbance (340 nm) was measured. All solutions were made in a 50 mM buffer, with the following reagent concentrations in the final mixture: 1.5 mM H_2_O_2_, 0.04 U glutathione peroxidase, 1 mM EDTA, 0.2 U glutathione reductase, 2 mM GSH, 0.04 U glutathione peroxidase, and 0.8 mM NADPH. Instead of the sample, a blank sample was created using the buffer, and the background was assessed (mixture containing studied sample and buffer only). In comparison to nmol of NADPH consumed/min in the blank (reagent) sample, one unit of enzyme activity was defined as nmol of NADPH consumed/min · mL sample [20].

##### Inhibition of Lipid Peroxidation 

The conjugated diene technique was used to measure antioxidant activity in the linoleic acid model system (linoleic acid oxidation test). Linoleic acid (800 mg) was freshly dissolved in pure MeOH (20 mL) and combined with 200 mL of 0.2 M sodium phosphate buffer (pH 6.5) and Tween 20. (6.5 mM conc. of Tween 20 was obtained). For 10 min, the emulsion was sonicated. A volume of 0.2 mL of the sample was combined with 1.8 mL of linoleic acid emulsion and incubated at 37 °C. After 4 h of incubation, samples (0.1 mL) were obtained and mixed with 1.2 mL of 100% MeOH. At 234 nm, the absorbance of a blank sample without an investigated solution was determined. The calibration curve was then created using ascorbic acid solutions (223.5–1676.3 µg ascorbic acid/mL) in the same way as the tested samples. The sample’s background was measured at 234 nm (a mixture containing studied sample and buffer only) [21].

##### β-Carotene Bleaching Test

β-carotene (7 mg) was combined with 350 mL linoleic acid and 2.8 g Tween 80 in 5 mL chloroform. Under vacuum (40 °C), chloroform was evaporated, and 100 mL of DDI water saturated with oxygen was added, followed by vigorous shaking. β-carotene/linoleic acid emulsion (200 µL) was combined with the sample (200 L). At 463 nm, the absorbance at zero time and the change in absorbance after 4 h at 50 °C were measured. Instead of the studied samples, a stock solution of ascorbic acid (0.94 mg/mL) was made, followed by a series of dilutions (9.4–94 µg/L), and utilized instead. The background of the samples was determined (a mixture containing the studied sample and DDI water only). The percent activity of samples was determined using blank samples containing only emulsion and DDI water [22].

To verify the statistical significance of the obtained results throughout antioxidant activity, the ANOVA test was used with the Statistica 12.0 software.

#### 2.2.6. Effect on Cholinesterase (ChE) Activity 

The colorimetric approach of Ellman [23] was employed, with the changes reported earlier [24]. After 5 min, the tested sample (10 µL) was combined with 20 µL of AChE (or BChE) solution (0.28 U/mL), 175 µL of 0.3 mmol/L DTNB (containing 10 mmol/L NaCl and 2 mmol/L MgCl2), and 110 µL of Tris-HCl buffer (50 mmol/L, pH 8.0) and completed with 35 µL of ATChI (or BTCh) (1.5 mmol/L). In place of the examined sample, samples containing 35 µL of Tris-HCl buffer were run in the same way (“blank” samples). Using “blank” samples containing ATCh (or BTCh) and DTNB completed to 345 µL with Tris-HCl buffer, the increase in absorbance owing to spontaneous hydrolysis of the substrate was observed. The “false-positive” effect of the examined compounds was measured according to Rhee et al. [25] with slight adjustments, as stated earlier [24]: the “false-positive” sample was left for incubation after mixing the substrate with the enzyme and buffer. Then an examined sample and DTNB were added, and the absorbance was measured immediately. 

The results were calculated using reference cholinesterase inhibitors (eserine, neostigmine, magniflorine, rivastigmine and donepezil). For this, 16 dilutions in pure DMSO (2.57–41.14 µg/mL) were made for each chemical. These solutions (10 µL) were tested and calibration curves were created as mentioned above.

All solutions utilized in a series of assays were produced in the same buffer, and each sample was evaluated at least eight times. The background of the sample (10 µL mixed with 365 µL of Tris buffer) was measured at 405 nm for calculations and removed. The test sample’s absorbance was then subtracted from the “blank” sample’s absorbance.

#### 2.2.7. Anti-Inflammatory Activity 

##### Anti-Hyaluronidase Activity 

The hyaluronidase inhibition was determined by a turbidimetric method described by Studzińska-Sroka et al. [26]. Twenty-five µL enzyme (30 U/mL of acetate buffer pH 7.0), 25 µL acetate buffer (50 mM, pH 7.0, with 77 mM NaCl and 1 mg/mL of albumin), 15 µL acetate buffer (pH 4.5), and 10 µL extracts were mixed, and incubated for 10 min at 37 °C. Then, 25 µL HA (0.3 mg/mL of acetate buffer pH 4.5) was added and incubated for 45 min at 37 °C. The undigested HA was precipitated with the addition of 200 µL 2.5% CTAB in 2% NaOH (pH 12). The mixture was incubated for 10 min at room temperature. The reaction mixture turbidance was measured as the absorbance at λ = 600 nm. Three independent experiments were carried out in triplicate, obtaining 9 independent results. The inhibition percentage was calculated by using the following equation:

$$\%\;inhibition\;activity=\frac{T_{S}-T_{C}}{T_{H}-T_{C}}\times 100\%$$

where *T_S_*—absorbance of the enzyme + HA + extract, *T_C_*—absorbance of the enzyme + HA, and *T_H_*—absorbance of the HA + extract.

The results are expressed as IC_50_ values, which corresponds to the extract concentration required for 50% of hyaluronidase inhibition. To verify the statistical significance of the obtained results, the ANOVA test was implemented with the Statistica 12.0 software.

##### Effect on Cyclooxygenase-2 (COX-2) Activity 

For the assay, chemicals from the Cayman COX-2 Assay Kit were prepared according to the manufacturer’s instructions and mixed with COX-2 enzyme (Human recombinant, Cayman No. 60122, pre-diluted 100-fold using 100 mM, pH 8.0 Tris buffer). A volume of 0.01 mL of the examined sample was combined with 0.12 mL of Tris buffer (100 mM, pH 8.0), 0.01 mL hemin, and shaken for 5 min at 25 °C before adding 0.02 mL colorimetric substrate and 0.02 mL arachidonic acid solution. Then, 0.02 mL COX-2 solution was added to start the reaction. The increase in absorbance during the room temperature incubation was measured at 590 nm. Simultaneously, a negative (blank) sample (buffer instead of the examined sample) and a positive sample (COX-2 inhibitor DuP-697) were run. The background of the examined samples (0.04 mL sample + 0.19 mL buffer) was also measured and accounted for in the calculations. Each sample was tested at least four times. The percentage of inhibition of enzyme activity was calculated (indicated by how many percent the activity was reduced in relation to the negative-blank sample for which the maximum activity was assumed as 100%, under the conditions used in the method). In addition, enzyme inhibition was expressed as acetylsalicylic acid equivalent concentration (mg/cm^3^) in the samples. Acetylsalicylic acid solutions were produced at 14 concentrations (0.2–10 mg/cm^3^) for this purpose and examined in the same way as the tested samples. 

#### 2.2.8. Microbiological Activity

##### Inoculum Standardization

For approximately 16 h, all microorganism strains were inoculated in Müeller-Hinton broth (pH 7.4). Using a spectrophotometer, the concentration of the suspensions were adjusted to 0.5 (optical density).

##### Assay of Antibacterial Activity Using Agar Well Diffusion Method

The Agar well diffusion method [27] was used to assess the antimicrobial activity of the crude and solvent extracts. Twenty mL of sterilized nutrient agar was put in sterile petri dishes. Using sterilized spreaders, 100 μL of standardized inoculate of each isolate was inoculated on nutrient agar plates after solidification. Using a sterile gel puncher with a 6 mm diameter, the wells were punched over the agar plates. Then, 100 μl of each extract were put in separate wells. The extracts were dissolved in 0.9% (*v*/*v*) NaCl, which served as a solvent extract negative control. Aqueous and extract concentrations in four distinct concentrations were examined. The plates were incubated for 24 h at 37 °C. To ensure dependability, triplets of the experiment were kept for each microorganism strain. The diameter of circular inhibitory zones produced around each well was measured in mm and recorded after incubation. 

## 3. Results and Discussion

The search for new varieties of blackberry leaves is extremely necessary because of their unique properties. The experimental property screening studies of four Rubi folium varieties were divided into two areas: (1) evaluation of the composition of the obtained extracts and identification of compounds responsible for their biological activity, and (2) screening of the extracts’ pharmacological activity.

**Figure 1:**
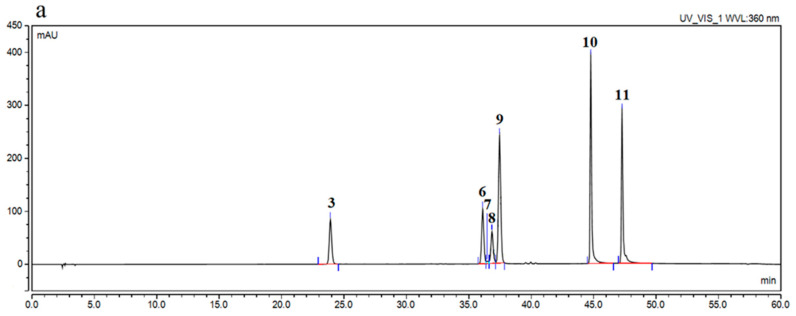

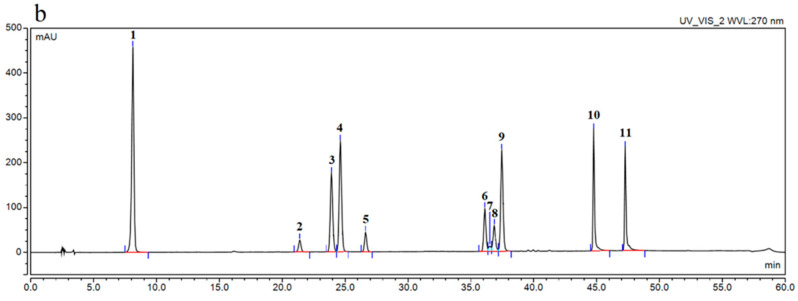
Chromatogram of phenolic compounds identified in blackberry leaves Loch Tay extracts: 1—gallic acid, 2—catechin, 3—caffeic acid, 4—syringic acid, 5—epicatechin, 6—rutin, 7—ellagic acid, 8—hyperoside, 9—isoquercetin, 10—quercetin, 11—kaempferol, at 360 nm (**a**) and 270 nm (**b**).

Due to the different solubilities of active compounds in inorganic and organic solvents, two types of extracts, aqueous and hydroalcoholic, were prepared. Most of the active compounds are freely soluble in ethanol, hence the choice of methanol and distilled water (7:3 *v*/*v*) as the extraction mixture, while the second type is aqueous extracts because they are a simple, economic and ecological alternative to organic solvents. To identify and determine active compounds contained in plant material, a high-performance liquid chromatography method supported by a photodiode array detector was developed. The developed HPLC-DAD method confirmed the presence of selected phenolic compounds in blackberry extracts (Figure 1). The retention times of the selected peaks were compared with the retention times of the reference substances, as well as their UV spectra. Seven of eleven compounds identified in extracts were determined quantitatively in the prepared aqueous and hydroalcoholic extracts; caffeic acid, ellagic acid, epicatechin at 270 nm, whereas quercetin, kaemferol, rutin and hyperoside at 360 nm. Validation parameters for each standard are presented in Tables S1 and S2 in Supplementary Material. The content of active compounds is presented in Table 2.

Hyperoside and rutin were present in all investigated samples analyzed by the HPLC-DAD method; these compounds, as poorly water-soluble, were obviously present in a greater amount in the hydroalcoholic extracts than the aqueous ones (Table 2). Detailed characteristics of the content of polyphenolic compounds of the described 4 varieties of blackberry leaves were reported for the first time in the presented studies; the species with the highest content of these compounds was blackberry leaves Loch Tay. Compared to other species described in the literature, the tested species contained more hyperoside in comparison with methanol extracts of *R. fructicosus* (0.70%), *R. caesius* (0.46%), *R. nessensis* (1.05%), *R. odoratus* (0.60%), *R. fructicosus* Gazda (0.82%), and *R. fructicosus* Thomfree (0.50%) [28]. 

Ellagic acid can occur in plants in the free form but it can also be in the form of ellagitannins from which it is released by hydrolysis. It may explain the much lower content of ellagic acid in the analyzed species compared to the data in the literature in methanol extracts after hydrochloric acid hydrolysis (extract concentration 2.0 mg/mL) of *R. fructicosus*—4.32%, *R. caesius*—4.15%, *R. nessensis*—6.89%, *R. odoratus*—3.76%, *R. fructicosus* Gazda—2.93%, and *R. fructicosus* Thomfree—4.21% [28].

The screening of biological effects of blackberry leaves extract was carried out in relation to (1) antioxidant, (2) anticholinesterase, (3) anti-inflammatory and (4) microbiological and properties.

It is advisable to utilize more than one antioxidant assay for determining the antioxidant activity of natural antioxidants in order to have a comprehensive understanding of the antioxidant properties of compounds and/or extracts. Therefore, in the presented study several methods expressing various aspects of the antioxidant action of polyphenols were used to provide a broader view of the antioxidant potential of blackberry leaves’ extracts. The DPPH, ABST, ORAC and SOD methods measure the ability of an antioxidant to scavenge free radicals, which is the most physiologically important mechanism of antioxidant activity. The CUPRAC, FRAP and HORAC methods measure the metal-chelating activity of antioxidants and hence indicate the compounds protecting ability against formation of hydroxyl radical. Additionally, enzymes such as superoxide dismutase (SOD), glutathione peroxidase (GPx), and glutathione reductase (GR) are components of the cell’s defense system to protect against oxidative damage, so they are useful models for assessing free radical scavenging by plant antioxidants.

Total phenolic content (TPC) was determined using the Folin–Ciocalteu method; obviously, a statistically higher content was found for hydroalcoholic extracts compared to water extracts (Table 3, extract concentration 10 mg/mL). Loch Tay species showed the highest TPC among 4 tested species. The obtained phosphate extracts (2 g of leaf samples extracted with 15 mL of phosphate buffer (75 mM, pH 7.0) using homogenizer; extract concentration 133.3 mg/mL) exhibited lower TPC (blackberry Chester Thornless—82.8 mg/g, Hull Thornless—74.0 mg/g and Triple Crown—91.0 mg/g) comparing to those tested in the presented work [29]. Moreover, investigations whether leaf age might have an effect on TPC, previous studies have shown that TPC is much higher in young leaves (from the upper part of shoots or stems) compared to older ones (from the lower part of shoots or stems) [29]. Polyphenolic composition and its antioxidant activity was described for blackberry pomace Chester (Soxhlet extractor, with 80% ethanol (*v*/*v*)), and it showed lower TPC compared to the leaf extracts tested in this study [30].

The TPC is highly correlated with antioxidant activity. Obviously, hydroalcoholic extracts exhibited higher antioxidant activity compared to water ones, due to higher TPC and higher individual polyphenolic compounds’ content. The highest antioxidant activity exhibited Loch Tay species, which was confirmed by 4 independent methods (Table 3 and Table 4). Comparing water extracts with those obtained previously (extract concentration 20.0 mg/mL), TPC for blackberry leaves was 75.4 and antioxidant activity obtained using DPPH was 125.2 and FRAP—36.7. Comparing those results with water green tea extract, known as a rich antioxidant source (TPC—84.8, DPPH—175.2, FRAP—47.0) [31], it could be concluded that blackberry Loch Tay leaves water extract also shows very good antioxidant activity and could be used as an antioxidant agent. Testing the ability to inhibit GP and GPx (Table 4), we noticed that the differences in activity between cultivars were small and statistically insignificant, therefore it was not possible to select the best cultivars on the basis of these studies alone; however, the results showed the high antioxidant potential of the extracts.

The fact that ellagic acid, hyperoside and epicatechin were detected in the extracts in the highest amounts, also guarantees the antioxidant activity of the extracts, both for the water and alcohol-based cases. Ellagic acid exhibits antioxidant, anti-mutagenic, anti-inflamatory as well cardioprotective properties [32]. In addition to the significant scavenging of DPPH free radicals by ellagic acid, it also inhibited the lipid peroxides production in V79-4 cells exposed to hydrogen peroxide. Ellagic acid has also been found to increase the activity of the three antioxidant enzymes SOD, CAT and GPX, which are altered in various diseases involving free radicals [33]. Hyperoside is also believed to be effective in protecting cells against oxidative stress through the induction of HO-1. Additionally, it elevated Nrf2 levels and its antioxidant response element binding activity was modulated by pre-ERK; also it activated ERK and restored cell viability that had been reduced by hydrogen peroxide [34]. Catechin and epicatechin, included to flavon-3-ols, are antioxidants which influence plasma antioxidant biomarkers and energy metabolism [35].

**Figure 2:**
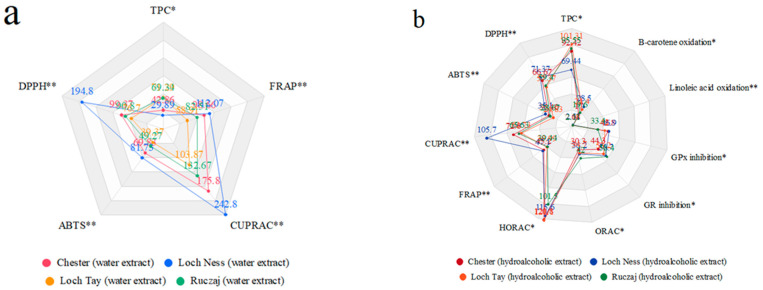
TPC in correlation with summarized antioxidant activity of blackberry leaves water (**a**) and hydroalcoholic (**b**) extracts. * the higher the value, the higher the activity. ** the lower the value, the higher the activity.

All the antioxidant activity data were summarized in the spider chart in Figure 2a,b. The data clearly show the outstanding antioxidant properties of the Loch Tay variety.

Polyphenols can operate as antioxidants, in addition to their traditional antioxidant activity, by influencing intracellular redox balance through alternative methods such as inhibiting pro-oxidative enzymes such as lipoxygenase. Such effects could point to the use of blackberry leaf extracts in the prevention of neurodegenerative illnesses on a case-by-case basis. The presented study showed the ability to inhibit butyrylcholinesterase (BChE) by Loch Ness hydroalcoholic extracts (Table 5). Phenolic acids and flavonoids are known compounds with anticholinesterase activity [36,37], which confirms the extract activity. Therefore, the use of blackberry leaves is suggested to prevent neurodegenerative diseases such as Alzheimer’s disease [4].

In addition to the antioxidant activity of the polyphenols present in blackberry, there are reports on its anti-inflammatory activity, expressed by inhibiting the hyaluronidase as well as cyclooxygenase activity [38]. Hyaluronidases are known as pro-inflammatory agents; hence, hyaluronidase degradation appears critical and imperative in several pathological conditions [39]. The anti-hyaluronidase activity was measured for all 4 species of water extracts. Due to the specifics of the study, the experiment could not be performed for the hydroalcoholic extracts. The highest activity was demonstrated by Loch Tay and Ruczaj varieties, and the difference between those two was not statistically significant (Table 6). There are no literature data on the anti-hyaluronidase activity of other blackberry leaves varieties; hence it is not possible to compare the results with the previous ones. COX-2 inhibition was similar in the case of all tested variants (Table 6).

Again, the most abundant compounds in the extracts, ellagic acid, hyperoside and epicatechin, are suspected of having anti-inflammatory effects, as confirmed in previous studies involving standards. Ellagic acid has been assigned as an anti-inflammatory agent that acts on several inflammatory mediators [40], including concentration-dependent inhibition of the 12-lipoxygenase isoform [41]. Also, quercetin derivatives i.e., rutin, hyperoside and isoquercetin, show similar properties [42]. Hyperoside has been reported to be anti-inflammatory by inhibiting arachidonic acid-induced edema and croton oil-induced edema. It also inhibited the COX-2 and hyaluronidase enzymes and suppressed the production of IL-6, TNF and NO in the peritoneal macrophages of LPS-stimulated mice [43]. Moreover, many in vitro and in vivo studies using various tissues confirm the anti-inflammatory effects of epicatechin by reducing activation of the NF-κB signaling pathway [44].

**Figure 3:**
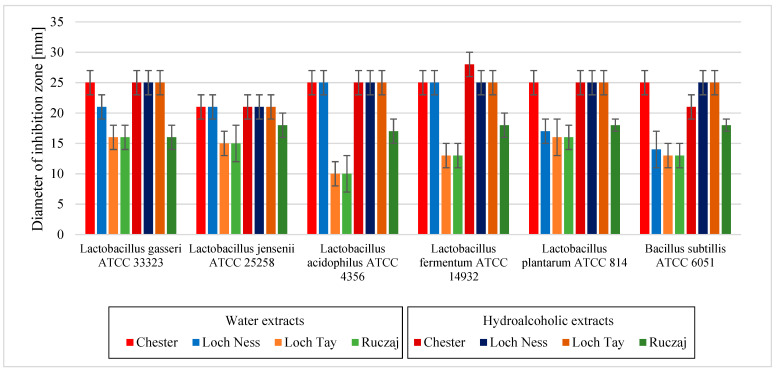
Microbiological activity of blackberry leaves extracts in their initial concentrations on health-promoting bacteria. Error bars define the SD values.

**Figure 4:**
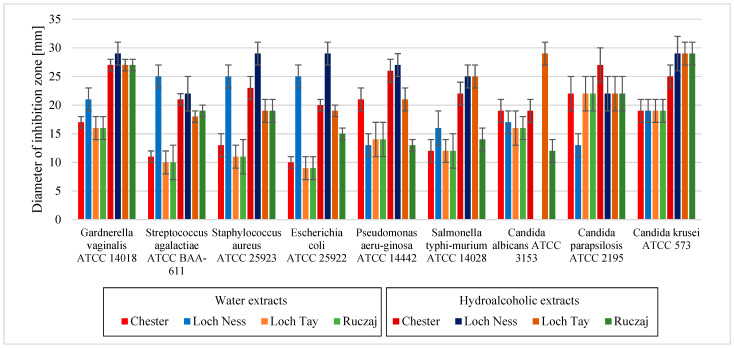
Microbiological activity of blackberry leaves extracts in their initial concentrations on potentially pathogenic microorganisms. Error bars define the SD values.

The last stage of the research involved the assessment of the influence of blackberry leaves extracts on microorganisms. The antimicrobial effect of blackberry leaves’ extracts was determined by inhibition of growth for both health-promoting (*Lactobacillus* spp. and *Bacillus* spp.) and potentially pathogenic microorganisms (*G. vaginalis, S. agalactiae, S. aureus, E. coli, P. aeruginosa, S. typhimurium* and *Candida* spp.) with extensive review of the activity for bacteria with a division for those living in the digestive tract (e.g., *Lactobacillus* spp. and *E. coli*), genital tract (e.g., *G. vaginalis* and *S. agalactiae*), on the skin (e.g., *S. aureus*), and also those that contribute to the formation of dental plaque and other oral diseases (e.g., *Candida* spp. and *S. aureus*). The microbiological activity of berries has been proven in past years, which was also demonstrated in this study [45,46,47]. So far, no similar studies have been carried out with the use of the studied varieties; therefore, it is difficult to refer directly to the literature data. Obviously, higher activity was obtained in the case of the hydroalcoholic extracts than in the water extracts (Figure 3 and Figure 4). Microbiological activity is widely associated with polyphenols’ strong anti-microbiological properties, which are also present in blackberry leaves extracts [48,49,50]. The lower activity of the Loch Tay and Ruczaj cultivars against probiotic bacteria (*Lactobacillus* spp.) confirms the hypothesis about the possible modification of the bacterial profile by blueberries. This is possible by increasing the number of beneficial bacteria and thereby improving gut health, which has also been proven for blackberries [45]. The potential of anthocyanin-rich blueberry extract has also been shown to reduce the adhesion of many pathogenic bacteria while reducing the infection probability [46]. Also, in this study, the blackberry leaves extracts showed a strong inhibition of e.g., *E. coli* or *Candida* spp. growth, the strongest in the case of the Loch Ness variety, which may suggest their extraordinary usefulness in inhibiting the infections’ development both in the gastrointestinal tract and in the oral cavity. In an earlier study by González et al., the antibacterial effects of blackberry extract against *P. gingivalis, F. nucleatum* and *S. mutans* were demonstrated [51]. Combined with anti-plaque activity by inhibiting the *Candida* spp. adhesion and previously demonstrated strong antioxidant and anti-inflammatory properties, blackberry extracts are a promising method for the prevention and/or treatment of periodontal infections.

## 4. Conclusions

It was found that the content of the analyzed phytochemicals in blackberry leaf extracts depends on the type of extract, which is related to the solubility of individual components. Research has shown the comprehensive antioxidant activity of extracts, based on both free radicals’ scavenging and metal-chelating activity; anti-inflammatory and antibacterial activity have also been proven. Based on numerous studies, the biological activity of all the tested varieties has been demonstrated to be broad, while the Loch Tay variety has been selected as the one with the greatest potential for biological activity, and its use in medicine may be further investigated. It can be assumed that the use of the leaves will be as beneficial to health as the consumption of fruit while reducing production costs and used as agri-food waste. Therefore, blackberry leaves may be a valuable new functional food as well as a source for development of new pharmaceutical formulations with standardized extracts.

## Figures and Tables

**Table 1:** An overview of the distribution of polyphenols compounds [2].

	Compound Name
Phenolic Acids	Neo-chlorogenic acid Caffeic acid Gallic acid p-coumaric acid Ellagic acid
Flavonols	Quercetin Quercetin-3-O-galactoside, hyperoside Quercetin-3-O-glucuronide, miquelianin Kaempferol
Flavan-3-ols	Catechin Epicatechin Epicatechin gallate methyl gallate
Ellagitannins	Sanguiin H-6 /Lambertianin C Casuarinin
Anthocyanins	Cyanidin-3-O-glucoside

**Table 2:** The content of phenolic compounds in blackberry leaves aqueous and hydroalcoholic extracts.

	Varieties	Chester		Loch Ness		Loch Tay		Ruczaj	
		Water Extract	Hydroalcoholic Extract	Water Extract	Hydroalcoholic Extract	Water Extract	Hydroalcoholic Extract	Water Extract	Hydroalcoholic Extract
Phenolic Compound		Content (µg/g Plant Material)							
*Phenolic acids*									
caffeic acid		18.35 ± 1.57	17.12 ± 0.81	20.65 ± 0.46	1.77 ± 0.03	606.62 ± 4.22	255.25 ± 6.62	85.89 ± 5.15	55.86 ± 6.59
ellagic acid		93.65 ± 9.48	515.30 ± 10.69	338.29 ± 10.89	703.78 ± 13.97	468.33 ± 5.17	783.06 ± 21.08	650.65 ± 11.17	876.82 ± 18.97
*Flavonols*									
quercetin		9.26 ± 0.42	23.61 ± 0.86	1.10 ± 0.24	15.98 ± 0.41	40.25 ± 0.89	45.79 ± 0.18	10.91 ± 0.78	30.25 ± 0.24
kaempferol		0.12 ± 0.05	0.72 ± 0.21	1.67 ± 0.29	2.12 ± 0.08	3.49 ± 0.27	4.46 ± 0.16	0.37 ± 0.07	1.69 ± 0.25
rutin		113.21 ± 1.46	117.09 ± 7.15	9.56 ± 0.80	28.69 ± 2.40	179.01 ± 11.03	204.12 ± 6.55	162.70 ± 9.52	445.21 ± 32.02
hyperoside		4723.72 ± 5.44	7094.32 ± 9.93	2234.92 ± 6.21	3775.87 ± 11.25	29,990.78 ± 14.07	30,854.28 ± 96.37	5969.87 ± 29.60	8047.17 ± 14.39
*Flavon-3-ols*									
epicatechin		35.07 ± 0.99	598.91 ± 16.76	4.67 ± 1.30	157.14 ± 8.94	416.04 ± 19.19	703.96 ± 4.28	77.03 ± 7.88	961.14 ± 29.40
		**content (% of dry weight)**							
*Phenolic acids*									
ellagic acid		0.01 ± 0.01	0.05 ± 0.01	0.03 ± 0.01	0.07 ± 0.01	0.05 ± 0.01	0.08 ± 0.01	0.07 ± 0.01	0.09 ± 0.01
*Flavonols*									
rutin		0.01 ± 0.01	0.01 ± 0.01	>0.01	>0.01	0.02 ± 0.01	0.02 ± 0.01	0.02 ± 0.01	0.04 ± 0.01
hyperoside		0.47 ± 0.01	0.71 ± 0.01	0.22 ± 0.01	0.38 ± 0.01	3.00 ± 0.01	3.09 ± 0.01	0.60 ± 0.01	0.80 ± 0.01
*Flavon-3-ols*									
epicatechin		>0.01	0.06 ± 0.01	>0.01	0.02 ± 0.01	0.04 ± 0.01	0.07 ± 0.01	>0.01	0.10 ± 0.01

**Table 3:** Antioxidant activity of blackberry leaves aqueous and hydroalcoholic extracts.

	TPC (mg GAE/g) *	DPPH IC_50_ (μg/mL)	ABTS IC_50_ (μg/mL)	CUPRAC IC_0.5_ (μg/mL)	FRAP IC_0.5_ (μg/mL)	HORAC Equivalent Gallic Acid Concentration (μg/cm^3^)	ORAC Equivalent Trolox Concentration (μM)
*Water extracts*							
Chester	42.86 ± 0.71	99.37 ± 2.47	69.83 ± 1.15	175.80 ± 9.23	98.66 ± 2.60	n/a	n/a
Loch Ness	29.89 ± 0.19	194.8 ± 5.59	81.73 ± 2.31	242.80 ± 3.12	112.07 ±3.49	n/a	n/a
Loch Tay	**71.29 ± 2.67**	**76.70 ± 3.92**	**39.37 ± 1.82**	**103.87 ± 11.94**	**58.91 ± 1.80**	n/a	n/a
Ruczaj	69.34 ± 1.89	90.80 ± 1.31	49.27 ± 1.21	132.67 ± 7.94	82.51 ± 4.20	n/a	n/a
*Hydroalcoholic extracts*							
Chester	92.42 ± 1.14	66.67 ± 1.27	30.40 ± 2.15	72.80 ± 3.16	45.20 ± 0.63	120.80 ± 8.20	30.40 ± 3.10
Loch Ness	69.44 ± 3.80	71.37 ± 2.19	35.10 ± 1.32	105.7 ± 3.89	47.10 ± 3.10	115.60 ± 10.3	36.20 ± 1.00
Loch Tay	**101.31 ± 0.11**	**57.37 ± 3.61**	**24.83 ± 0.23**	**62.73 ± 3.89**	**39.99 ± 0.58**	**121.10 ± 11.2**	34.70 ± 2.20
Ruczaj	95.55 ± 2.09	59.4 ± 0.87	28.67 ± 1.72	65.63 ± 2.91	39.44 ± 1.61	101.50 ± 4.20	**42.00 ± 2.40**

Data expressed as mean ± SD. * Data expressed as miligrams of gallic acid equivalents (GAE) per 1g of plant material. The best values (the TPC and the lowest IC_50_ or IC_0.5_) are shown in bold. n/a—non applicable.

**Table 4:** Antioxidant activity of blackberry leaves hydroalcoholic extracts.

	Effect on SOD Activity		Effect on GR and GPx Activity			Linoleic Acid Oxidation	β-Carotene Oxidation
	Enzyme Inhibition (%)	GR Inhibition (%)	GR Inhibitory Activity (μmol Consumed NADPH/min Incubation)	GPx Inhibition (%)	GPx Inhibitory Activity (nmol Consumed NADPH/min Incubation)	Equivalent Ascorbic Acid Concentration (mg/mL)	Equivalent Ascorbic Acid Concentration (µg/mL)
*Hydroalcoholic extracts*							
Chester	18.3 ± 1.5	44.3 ± 3.2	1.7 ± 0.2	**45.8 ± 3.2**	91.3 ± 4.6	**2.04 ± 0.12**	**17.0 ± 3.2**
Loch Ness	**30.2 ± 2.5**	**56.7 ± 2.1**	2.2 ± 0.1	**46.9 ± 2.4**	93.5 ± 3.5	2.51 ± 0.04	28.5 ± 1.7
Loch Tay	**27.6 ± 2.0**	53.2 ± 3.1	2.1 ± 0.3	42.0 ± 3.3	83.7 ± 4.7	**2.18 ± 0.17**	24.4 ± 2.1
Ruczaj	16.4 ± 4.5	**58.4 ± 1.8**	2.3 ± 0.3	33.4 ± 1.2	66.6 ± 2.1	**2.11 ± 0.06**	**19.6 ± 1.8**

Data expressed as mean ± SD. The best values are shown in bold.

**Table 5:** Effect on BChE activity.

Sample	Equivalent Reference Concentration (μg/mL)				
	Neostigmine	Magniflorine	Donepezil	Eserine	Rivastigmine
*Hydroalcoholic extract*					
Loch Ness	2.2 ± 0.1	6.9 ± 0.1	1.2 ± 0.0	1.4 ± 0.1	11.2 ± 0.1

**Table 6:** Anti-inflammatory activity of blackberry leaves extracts.

	Anti-Hyaluronidase Activity		Effect on COX-2 Activity	
	IC_50_ (μg/mL)		Equivalent Acetylsalicylic Acid Concentration (mg/cm^3^)	COX-2 Inhibition (%)
*Water extracts*		Hydroalcoholic extract		
Chester	160.69 ± 15.20	Chester	3.23 ± 0.1	**84.6 ± 3.5**
Loch Ness	180.09 ± 9.14	Loch Ness	3.22 ± 0.1	**82.1 ± 3.2**
Loch Tay	**129.30 ± 3.27**	Loch Tay	3.22 ± 0.0	**82.1 ± 2.0**
Ruczaj	**127.36 ± 4.13**	Ruczaj	3.23 ± 0.0	**84.6 ± 1.6**

Data expressed as mean ± SD. The best values (the lowest IC_50_ and the highest COX-2 inhibition) are shown in bold.

## Data Availability

The data is contained within the article or supplementary material.

## Supplementary Materials

The following are available online at https://www.mdpi.com/article/10.3390/antiox10121945/s1, Table S1: Validation parameters for standard detected at 270 nm, Table S2: Validation parameters for standard detected at 360 nm.
