# Supplementary material for: Blackberry Leaves as New Functional Food? Screening Antioxidant, Anti-Inflammatory and Microbiological Activities in Correlation with Phytochemical Analysis
Source: Antioxidants (Basel). 2021 Dec 4;10(12):1945. doi: 10.3390/antiox10121945 (PMC8750396; doi:10.3390/antiox10121945)
Supplement: Supplementary file 1 [file antioxidants-10-01945-s001.zip › antioxidants-1479293-supplementary.pdf]

Table S1. Validation parameters for standard detected at 270 nm

	Phenolic acids		*Flavonols*				*Flavon-3-ols*
	caffeic acid	ellagic acid	quercetin	kaempferol	rutin	hyperoside	epicatechin
Linearity: y = *a*x + *b*							
$a \pm S_a$	0.1418 ± 0.0051	0.1942 ± 0.0062	0.2005 ± 0.0135	0.2368 ± 0.0134	0.2463 ± 0.0148	0.0509 ± 0.0022	0.0512 ± 0.0028
$b \pm S_b$	insignificant (α=0.05)	insignificant (α=0.05)	insignificant (α=0.05)	insignificant (α=0.05)	insignificant (α=0.05)	insignificant (α=0.05)	insignificant (α=0.05)
Correlation coefficient (*r*)	0.9999	0.9999	0.9995	0.9996	0.9996	0.9998	0.9996
Range of linearity [μg/mL]	460.00 - 2300.00	240.0 - 1200.0	260.00–1300.00	200.00–1000.00	220.0 - 1100.00	200.00–1000.00	240.00 - 1200.00
Limit of detection (LOD) [μg/mL]	54.0708	25.0042	57.3062	37.2537	43.4250	28.2275	43.5613
Limit of quantification (LOQ) [μg/mL]	163.8508	75.7702	173.6552	112.8899	131.5909	85.5378	132.0040

S_a standard deviation of slope; S_b standard deviation of intercept, *t*, calculated values of Student's t test, $t_{\alpha,f}$ = 2.228 critical values of Student's test for degrees of freedom *f* = 10 and significance level α = 0.05.

Table S2. Validation parameters for standard detected at 360 nm

	Phenolic acids		*Flavonols*			
	caffeic acid	ellagic acid	quercetin	kaempferol	rutin	hyperoside
Linearity: y = *a*x + *b*						
$a \pm S_a$	0.0674 ± 0.0031	0.0780 ± 0.0011	0.2877 ± 0.0117	0.2755 ± 0.0150	0.2711 ± 0.0172	0.0551 ± 0.0024
$b \pm S_b$	insignificant (α=0.05)	insignificant (α=0.05)	insignificant (α=0.05)	insignificant (α=0.05)	insignificant (α=0.05)	insignificant (α=0.05)
Correlation coefficient (*r*)	0.9997	0.99998	0.9998	0.9997	0.9995	0.9998
Range of linearity [μg/mL]	460.0 - 2300.1	240.0 - 1200.0	260.00–1300.00	200.00–1000.00	220.0 - 1100.0	200.00–1000.00
Limit of detection (LOD) [μg/mL]	70.3544	3.5426	34.5431	35.6583	45.6525	28.0352
Limit of quantification (LOQ) [μg/mL]	193.7049	10.6279	173.6552	108.0553	138.3408	84.9552

S_a standard deviation of slope; S_b standard deviation of intercept, *t*, calculated values of Student's t test, $t_{\alpha,f}$ = 2.228 critical values of Student's test for degrees of freedom *f* = 10 and significance level α = 0.05.
